# Effect of Engineered Biomaterials and Magnetite on Wastewater Treatment: Biogas and Kinetic Evaluation

**DOI:** 10.3390/polym13244323

**Published:** 2021-12-10

**Authors:** Gloria Amo-Duodu, Emmanuel Kweinor Tetteh, Sudesh Rathilal, Edward Kwaku Armah, Jeremiah Adedeji, Martha Noro Chollom, Maggie Chetty

**Affiliations:** Green Engineering and Sustainability Research Group, Department of Chemical Engineering, Faculty of Engineering and The Built Environment, Durban University of Technology, Durban 4001, South Africa; gamoduodu04@gmail.com (G.A.-D.); rathilals@dut.ac.za (S.R.); edwardkarmah@gmail.com (E.K.A.); jerry_4real@live.com (J.A.); mnchollom@gmail.com (M.N.C.); chettym@dut.ac.za (M.C.)

**Keywords:** anaerobic digestion, biosorbent, biostimulant, magnetite, nanoparticles, kinetic model

## Abstract

In this study, the principle of sustaining circular economy is presented as a way of recovering valuable resources from wastewater and utilizing its energy potential via anaerobic digestion (AD) of municipality wastewater. Biostimulation of the AD process was investigated to improve its treatability efficiency, biogas production, and kinetic stability. Addressing this together with agricultural waste such as eggshells (CE), banana peel (PB), and calcined banana peels (BI) were employed and compared to magnetite (Fe_3_O_4_) as biostimulation additives via 1 L biochemical methane potential tests. With a working volume of 0.8 L (charge with inoculum to substrate ratio of 3:5 *v*/*v*) and 1.5 g of the additives, each bioreactor was operated at a mesophilic temperature of 40 °C for 30 days while being compared to a control bioreactor. Scanning electron microscopy and energy dispersive X-ray (SEM/EDX) analysis was used to reveal the absorbent’s morphology at high magnification of 10 kx and surface pore size of 20.8 µm. The results showed over 70% biodegradation efficiency in removing the organic contaminants (chemical oxygen demand, color, and turbidity) as well as enhancing the biogas production. Among the setups, the bioreactor with Fe_3_O_4_ additives was found to be the most efficient, with an average daily biogas production of 40 mL/day and a cumulative yield of 1117 mL/day. The kinetic dynamics were evaluated with the cumulative biogas produced by each bioreactor via the first order modified Gompertz and Chen and Hashimoto kinetic models. The modified Gompertz model was found to be the most reliable, with good predictability.

## 1. Introduction

Today’s energy-intensive development has led to a surging demand for fossil fuels, which generate environmental pollution and impacts the ecosystem through global warming [1,2]. This has stimulated the search for alternative energy sources that are both sustainable and eco-friendly, to mitigate the environmental crisis [3,4,5]. Therefore, the exploration of cost-effective technology and sustainable energy resources in wastewater settings, to generate biogas to boost the water economy and its reclamation for reuse, has become important. In addition, the environmental challenge and cost involved in discharging biowaste (banana peels, eggshell, orange peels, sludge, etc.) [6,7], underpin its importance in being engineered as a biostimulant for wastewater treatment and biogas enhancement.

Generally, anaerobic digestion (AD) is considered as one of the most valuable techniques that converts the organic matter present in the biowaste to renewable energy in the form of methane (CH_4_)-enriched biogas [8,9,10]. When the bioreactor is run at optimal conditions, production of the bioenergy such as methane (60–70%) and stabilised digestate by AD creates economic opportunities and eases pollution [8,11,12]. The AD process utilizes microorganism degradation potential in an ecologically sustainable [13,14,15], odour-reducing, and pathogenic organism-degrading process, especially in reactors running at mesophilic (25–45 °C) and thermophilic (>45 °C) temperatures [14,15]. Furthermore, produced biogas often contains impurities such as H_2_S and CO_2_, which lower the calorific value of biogas and are detrimental to equipment like pipes and combustion engines [3,16]. Some of the intriguing techniques used in addressing this include co-digestion, pre-treatment, different designs of reactor configurations, and the use of additives to stimulate bacteria growth and prevent inhibitory effects [2,8,16,17,18,19].

In recent studies, micro and macro nutrients were found to stimulate methane production and sustain the AD process up to a critical concentration range, after which inhibition occurs [7,9,20]. Other researchers examined the impact of integrating one or two metals into anaerobic biogas production, whereas some elements may have antagonistic or synergistic effects [7,21,22]. Goli et al. [23] increased the production of biodiesel by comparing other homogenous and heterogenous calcium oxide (CaO) catalysts produced from chicken eggshell. Sridhar [24] also studied the use of both calcined and natural eggshell to remove heavy metals (Pb and Cu) from real automotive wastewater, where high efficiency was attained. Amo-Duodu et al. [25] reported 80% increase in biogas yield from sugar refinery wastewater by adding metallic nanoparticles (Fe, Ni, and Cu) at a hydraulic retention time of 10 days and mesophilic temperature of 40 °C. Despite the potential benefits of trace metals on biogas production [2,15,26] and wastewater treatment using the most prevalent materials such as activated carbon, alumina, and silica, [27,28], their extensive use is hampered by cost [29,30]. Therefore, exploring less expensive biomaterials as a source of nutrients and biostimulant for the AD process can make it more economically feasible.

Consequently, advancement of nanomaterials for wastewater treatment is associated with many roadblocks including regulatory challenges, technical hurdles, and public perception [4,31,32,33]. In addition, there are uncertainties about the impact of nanomaterials on the environment [34,35,36] and scarcity of comprehensive cost–benefit analyses, as compared to existing technologies [37,38]. In addressing these challenges, agro-wastes such as coconut shells, banana and orange peels, and eggshells are gaining attention for wastewater purification and adsorption as biochar or activated carbons [6,39]. Although agro-wastes are generated in large quantities annually, posing a threat to the environment, landfills can act as biomaterials [6,7,37]. Table 1 presents some reported materials used in wastewater treatment. For instance, banana peels have been used as absorbents in adsorption of heavy metals from wastewater [24,40]. Egg shell has been used in coagulation processes for the removal of heavy metals [23]. On the other hand, magnetite (Fe_3_O_4_) has been used as a biostimulant for biogas production [41].

South Africa is estimated to produce 54.2 million tons of waste (municipal, commercial, and industrial) annually [41,42]. About 10% of this is recovered and recycled for other purposes, while the remaining 90% is landfilled or discarded [41]. There is a pressing concern for exploring the possibility of improving AD biogas production via the addition of absorbents (banana peels and eggshells) and magnetic biochar (made up of banana peel and powered magnetite). Therefore, this study seeks to explore the feasibility of calcined banana peels (BI), uncalcined banana peels (PB), magnetite (Fe_3_O_4_), and eggshell (CE) to improve the biogas yield via biochemical methane potential (BMP) tests. In addition, the degree of degrading the organics in the wastewater was kinetically studied using the first order, modified Gompertz, and Chen and Hashimoto kinetic models to establish their performance.

## 2. Materials and Methods

### 2.1. Engineered Biomaterials and Characterisation Techniques

The raw eggs and bananas, purchased from a local South African market in Durban, KwaZulu Natal Province, were washed with distilled water. The assay of preparing biochar described by Li et al. [6] was followed. The crushed eggshells (CE) and banana peels (PB) were then oven dried at 80 ℃ for 24 h. The banana peels-based biochar (BI) was prepared from the dehydrated CE, by soaking 5 g CE in 50 mL 20% vol H_3_PO_4_ solution for 1 h [6]. This was further calcined at a furnace temperature of 550 ℃ for 1 h. The physical morphologies and elemental compositions of the biomaterials were analysed using scanning electron microscopy and energy dispersive X-ray (SEM/EDX) analysis. The samples were firstly sputter coated with carbon to do the analysis. This was outsourced by using the University of Cape Town, South Africa SEM/EDX equipment (Nova NanoSEM coupled with EDT and TLD detector) operated at an acceleration voltage of 5 kV with a magnification range of 10–50 k.

### 2.2. Synthesis and Characteristics of the Magnetite (Fe_3_O_4_)

The magnetite (Fe_3_O_4_) used in this study was prepared by following the co-precipitation assay by Tetteh and Rathilal [5]. The chemicals used included sodium hydroxide pellets (NaOH), ferrous sulphate heptahydrate (FeSO_4_·7H_2_O), oleic acid (surfactant), and ferrous chloride hexahydrates (FeCl_3_·6H_2_O), which were all analytical grade supplied by Sigma Aldrich, South Africa. Stock solutions of 0.4 M Fe^3+,^ and 0.2 M Fe^2+,^ were first prepared by weighing 108.12 g and 55.61 g respectively and dissolving them with 1 L deionized water. In addition, 3 M NaOH stock solution was prepared by dissolving 199.99 g of NaOH with 1 L deionized water. The magnetite (Fe_3_O_4_) was then prepared in the volume ratio of 1:1, by using 500 mL of Fe^3+^ and Fe^2+^ stock solutions each. To ensure homogeneity, the solution was then stirred on a magnetic hotplate continuously, while adding 4 mL of oleic acid dropwise. The pH of the solution was then adjusted to a pH of 12 with 250 mL of the 3M NaOH until black precipitate was formed. Afterwards, thick black precipitate was then heated (ageing) at 70 °C. The supernatant was decanted, and the precipitate washed thrice with distilled water and ethanol to get rid of any form of unwanted particles. An oven drying was carried out at 80 ℃ for 12 h and then furnace calcination of 550 ℃ for 1 h. Samples obtained were characterized via the Brunauer–Emmett–Teller (BET) analysis by using Micromeritics TriStar II Plus equipment (Durban, South Africa) coupled with Tristar Plus software version 3.01. The carrier gases used were helium and nitrogen. Prior to the analysis, the sample was degassed at a temperature of 400 °C for 24 h. It was allowed to cool and then kept under nitrogen gas at a pressure of 5 mmHg for 24 h. The magnetite surface area of 27.59 m^2^/g, pore volume of 0.008 cm^3^/g and pore size of 1.48 nm was achieved.

### 2.3. Wastewater and Inoculum Samples

The wastewater sample (substrate) was collected from a biofiltration sample point of a local South African municipality wastewater treatment plant in the KwaZulu-Natal province. The activated sludge sampled from the anaerobic digester point source was used as the inoculum. An inoculum to substrate ratio of 3:5 (volume basis) was used for each 1 L bio-digester. Standard methods for the examination of water and wastewater [43] were employed to characterise the substrate and inoculum in triplicates with the results shown in Table 2. Color and turbidity were analysed with the spectrophotometer (HACH DR3900, Hach Company, Loveland, CO, USA) and turbidity meter (HACH 210, Hach Company, Loveland, CO, USA), respectively. Using the COD high range vials (HACH), 0.2 mL of the samples were measured and poured into the COD vials. It was then digested at 150 °C for 2 h. After the digestion, the vials were cooled at room temperature and the COD was measured using the spectrophotometer (HACH DR3900, Hach Company, Loveland, CO, USA). 

### 2.4. Biochemical Methane Potential (BMP) Test

**Figure 1:**
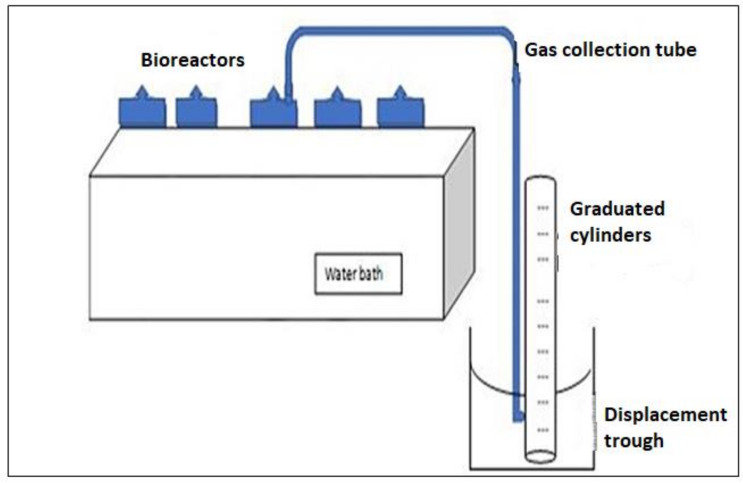
A biochemical methane potential (BMP) test set-up.

A lab-scale batchwise anaerobic system was setup for the BMP test and operated according to Hulsemann et al. [13]. In Figure 1, the BMP test setup consists of a biodigester, a biogas collecting system, and temperature-controlling (water bath) units. Five 1 L Schott bottles with a working volume of 800 mL were used as biodigesters, closed with Teflon caps (three ports). The outlet gas nozzle was connected to the biogas collecting unit via the downward displacement technique using a 1 L graduated cylinder placed upside down in another 5 L cylinder filled with water. Each bioreactor was charged with 1.5 g biomaterials, wastewater (500 mL), and sludge (300 mL) as presented in Table 3. Each bioreactor was purged with nitrogen gas for 2 min, to create the anaerobic conditions. To maintain the temperature at mesophilic conditions (40 ± 2.5 ℃) for 30 days, each bioreactor was immersed in the 20 L water bath (WBST0001, United Scientific, Cape town, South Africa) system. After recording the daily biogas produced, each bio-digester was manually shaken for 2 min. All the experiments were done in triplicates, and the results are averagely presented.

After 30 days of the experiment, the pH, COD, color, turbidity, TS and VS for each bioreactor was determined. The standard Equation (1) of calculating the efficiency of the contaminant removal was employed.

(1) $$Reactor\;efficiency=\left(\frac{C_{i}-C_{f}}{C_{i}}\right)\times 100$$

where $C_{i}$ = contaminant in the influent and $C_{f}$ = contaminant in the effluent. The cumulative biogas production data obtained was evaluated by first-order and modified Gompertz Equations (2) and (3), respectively.

### 2.5. Kinetic Study of BMP System

The quantitative comparison of biogas production during the batch mesophilic AD process with different biomaterials was modelled with first order (2), modified Gompertz (3) and Chen and Hashimoto (4) models adapted from Budiyono et al. [44] and Mu et al. [45].

(2) $$Y\left(t\right)=\mathrm{Ym}\;\left[1-\exp(-\mathrm{kt}\right)]$$

(3) $$Y\left(t\right)=\mathrm{Ym}.\exp\left(-\exp\left[\frac{2.7183R_{\max.}}{\mathrm{Ym}}\left[\lambda -t\right]\right]+1\right)$$

(4) $$Y\left(t\right)=\mathrm{Ym}\;\left(1-\frac{K_{\mathrm{CH}}}{\mathrm{HRT}\times R_{\max}+K_{\mathrm{CH}}-1}\right)$$

where

Y(t) is cumulative specific biogas yield (mL/g COD), 

Ym is the maximum biogas production (mL/g COD), 

λ is lag phase period or minimum time to produce biogas (days), 

t is the cumulative time for biogas production (days), 

k = R_max_/ym (1/day),

R_max_ is the maximum specific substrate uptake rate (mL/g COD.day),

k is a first-order rate constant (1/d) and

K_CH_ is the Chen and Hashimoto kinetic constant.

## 3. Results and Discussion

### 3.1. Surface Morphology

**Figure 2:**
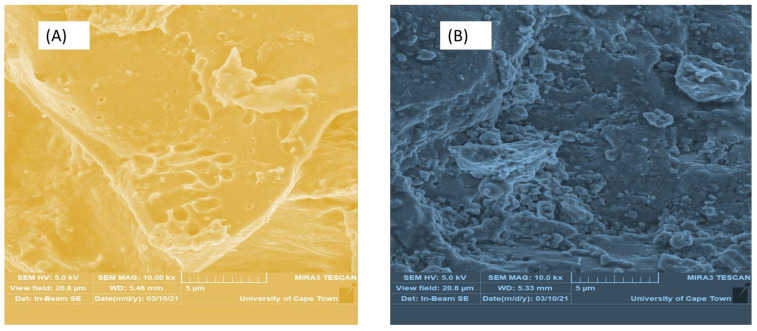

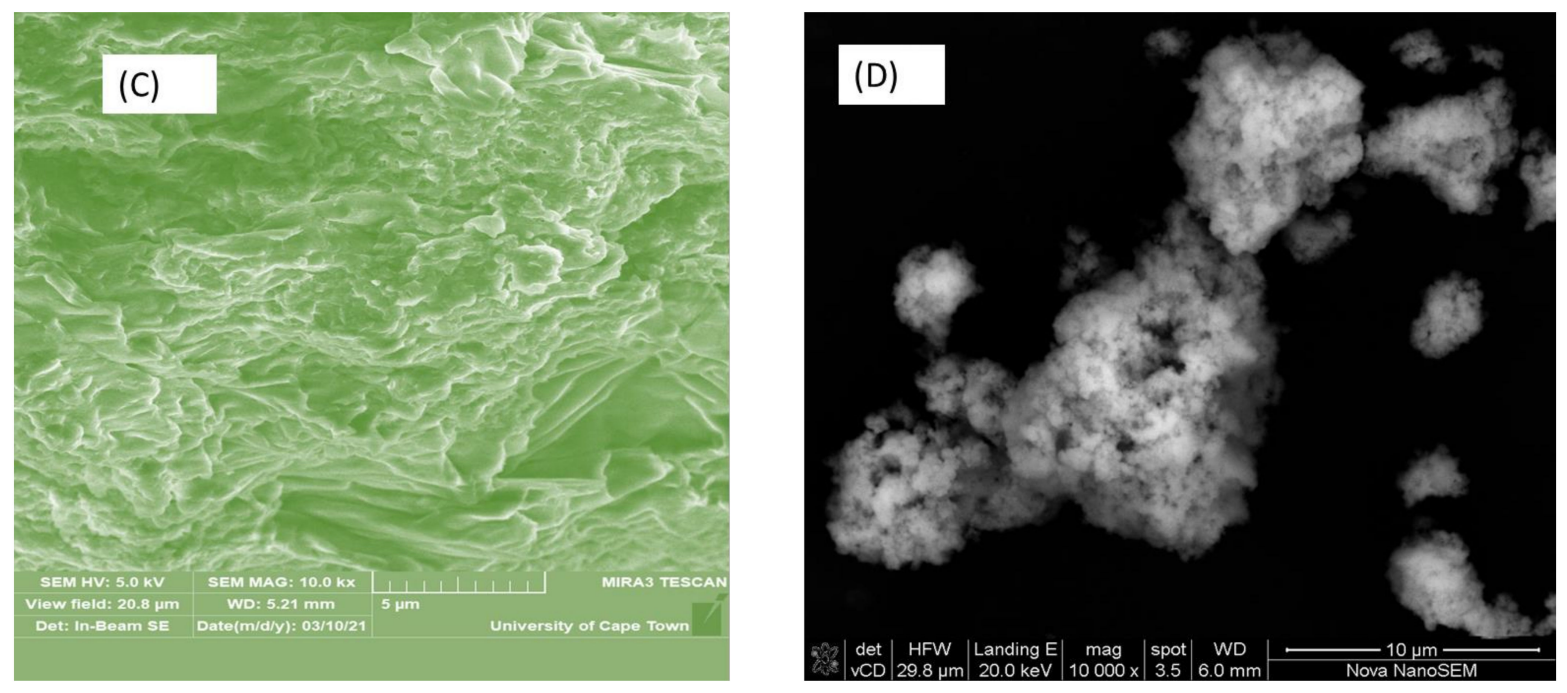
SEM images of biomaterials with view field of 20.8 µm at high magnification of 10 kx; (**A**) BI, (**B**) CE, (**C**) PB and (**D**) Fe_3_O_4_.

Figure 2 presents the scanned images of the biomaterials (A) BI- calcined banana peels, (B) CE-crushed egg shell, (C) PB-banana peels, and (D) magnetite (Fe_3_O_4_). The micrographs (Figure 2A–C) at 5 µm showed the same high magnification of 10 kx and view field of 20.8 µm. This revealed patchy fragmented surfaces with proportioned ridge-like strands of amorphous structures [40]. It was observed that the micrograph (Figure 2D) at the microscale of 10 µm and similar high magnification of 10 kx and view field of 20.8 µm had regular cellular structure. This makes the appearance of the magnetite (Figure 2D) different to that of the biomaterials (Figure 2A–C). The calcination temperature at 550 °C for 1 h changed the banana’s organics (BI) to produce brittle and flaky hydrochar, leaving residual concave plates (Figure 2A) to differ from the raw banana (PB) (Figure 2C). The surface area and porosity (6 mm) of the magnetite (Figure 2D) were found to be the highest, followed by BI (Figure 2A) of 5.48 mm as compared to PB (Figure 2C) of 5.21 mm and CE (Figure 2B) of 5.33 mm. The creation of a broad pore size distribution (Fe_3_O_4_ > BI > CE > PB), ranging from narrow microspores to wide mesopores, may be linked to the high calcination temperature (550 ℃), which improved the liquid–solid adsorption capacity [46]. This affirms treating PB with oxidizing solutions before calcination reduces amorphous cellulose transition [24] and therefore improves the surface adhesive properties by removing superfluous impurities from the rough surface [40,46].

**Figure 3:**
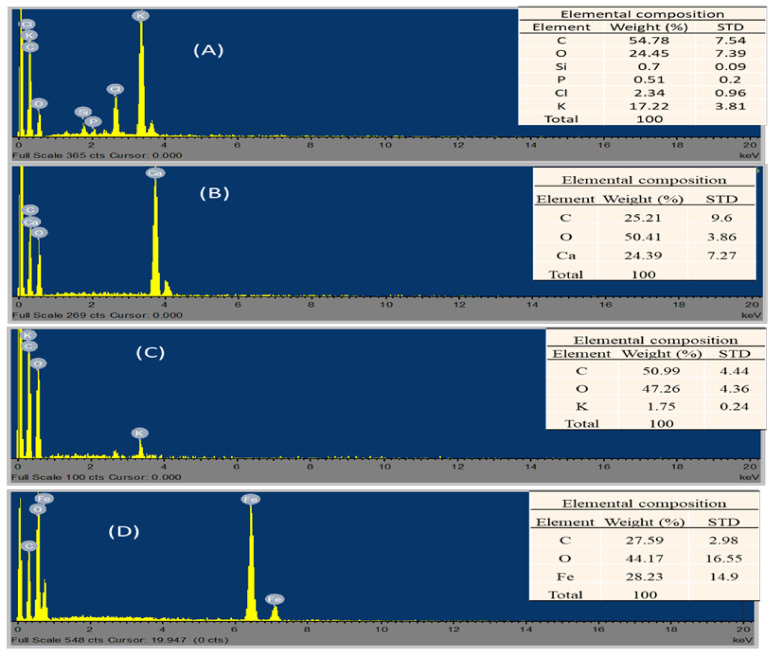
EDX spectrum images and tabulated elemental distribution of biomaterials with view field of 20.8 µm at high magnification of 20 kx. (**A**) BI, (**B**) CE, (**C**) PB and (**D**) Fe_3_O_4_.

Figure 3 presents the EDX spectrum with tabulated elemental distribution of (A) BI, (B) CE, (C) PB and (D) Fe_3_O_4_. This revealed that carbon (30–55% C) and oxygen (25–50% O) were the most dominant elements in all the biomaterials. Apparently, the presence of the carbon (C) in the magnetite (Figure 3D) EDX spectra was a result of the samples being coated under carbon gases. As revealed by the SEM image (Figure 2D) with bulbous morphology of the aggregates, suggests that the iron minerals were partly covered by carbon particles, which might be due to the calcination treatment and the carbon coating of the sample prior to analysis. Aside from that, CE (Figure 3B) was found to constitute calcium carbonate (25% CaCO_3_). Likewise, Fe_3_O_4_ (Figure 3D) had 28% Fe and the PB (Figure 3C) had 2% K. Conversely, the EDX and elemental composition were denatured in the calcined banana peel (BI), which revealed 17% K and 2% Cl as well as less than 1% P and 1% Si. The additional components in the BI could have increased its surface area, facilitating its biosorption and reusability [40].

### 3.2. Biodegradation Efficiency

**Figure 4:**
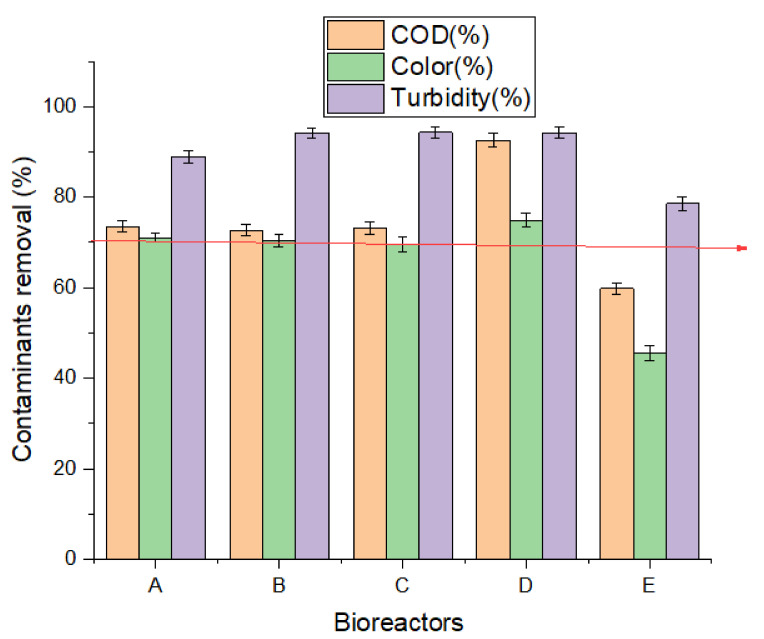
Biodegradation efficiency of bioreactors (A–E) for HRT of 30 days.

After 30 days of digestion, the degree of biodegradation was assessed based on the contaminant removal from the wastewater with respect to color, turbidity, and COD for each bioreactor. Figure 4 shows the effect of the biomaterials on the bioreactors (A–D) in removing above 70% contaminants as compared to that of the control bioreactor (E) with <70% efficiency. Bioreactor D showed a tremendous performance with contaminant removal of 92.59%, 74.86%, and 94.13% of COD, color, and turbidity removal, respectively. This was followed by bioreactor B with 72.69% COD, 70.35% color, and 94.13% turbidity > bioreactor C (73.11% COD, 69.65% color, and 94.26% turbidity) > bioreactor A (73.53% COD, 71.05% color, and 88.93% turbidity) > bioreactor E (59.83% COD, 45.61% color and 78.55% turbidity). This result supports that bioreactor D being charged with the Fe_3_O_4_ had high surface area and catalytic properties as revealed by the SEM/EDX result (Figure 2D). Evidently, the surface area and adsorptive capacity of the biomaterials (Figure 2A–C) also influenced the biodegradation performance of bioreactors A–C as compared to the control bioreactor E. This agrees with other researchers reporting that biomaterials have high catalytic properties, large surface areas, pore size, and good adsorption properties, all of which can influence treatability performance of wastewater [6,40]. In addition, eggshell and banana peels have been employed by other researchers for remediation of wastewater and removal of heavy metals, and were found to be successful [6,24,46], consistent with the current study.

### 3.3. Effect of Biosorbent on Biogas Yield

Results of the average and cumulative biogas yield obtained after the 30 days of digestion are summarised in Table 4. The increasing order of the bioreactors’ average biogas yield was found as follows: D (40 mL/day) > B (34 mL/day) > C (33 mL/day) > A (32 mL/day) > E (25 mL/day). Bioreactor D was found to be the most efficient, with an average daily biogas production of 40 mL/day and cumulative yield of 1117 mL/day. Thus, the presence of the Fe_3_O_4_ additives enriched the substrate nutrients, which stimulated the microbial activities of the methanogens to increase the biogas production [47,48]. Again, studies by other researchers have shown similar observations, where the addition of the Fe_3_O_4_ [49,50] increased biogas and methane yield, as compared to the control.

**Figure 5:**
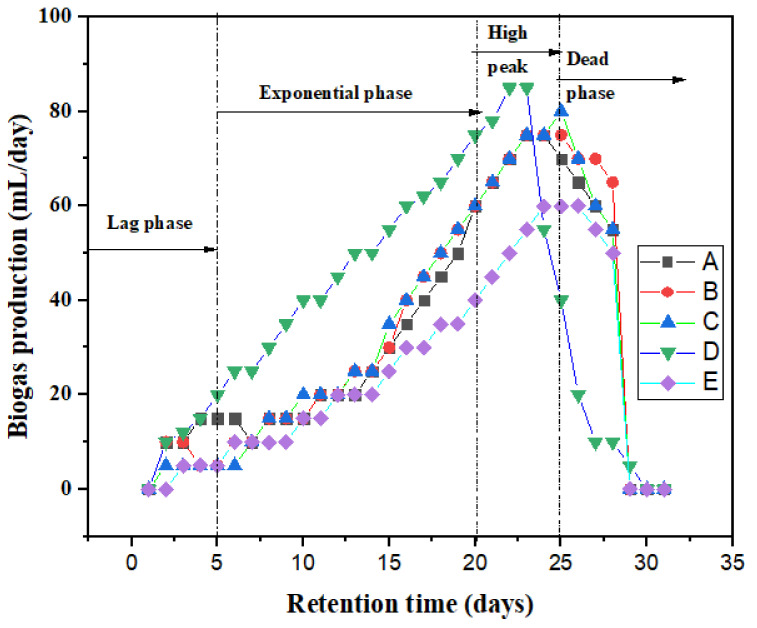
Daily biogas production of bioreactors (A–E) for HRT of 30 days.

Figure 5 presents the specific daily biogas production curve for the bioreactors (A–E). It was observed that during the first five days of the digestion process, the yield was below 20 mL/day which can be attributed to the fact that the microorganisms were still getting acclimatized to the environment (lag phase). From day 6 to 20, it was observed that there was a gradual increase in the biogas yield from 20 mL/day to about 80 mL/day for bioreactors A–D (exponential phase). All the bioreactors (A–E) attained maximum yield (high peak) between days 20–25, before yields started to decline from day 25 to 30 (death phase). From Table 1, comparing this study results to that of previous studies affirms that the use of biomaterials and magnetite can influence the AD process for biogas production and wastewater remediation [50].

### 3.4. Kinetic Model of the Biogas Production

**Figure 6:**
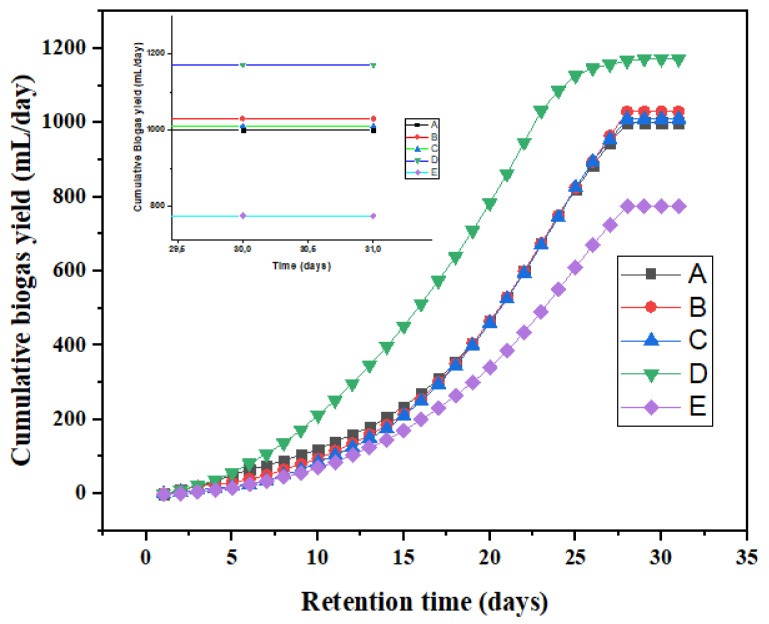
Cumulative biogas yield for bioreactors A–E for HRT of 30 days.

The kinetic study was carried out to ascertain the impact of the kinetic dynamics of the biomaterials in the AD process. To evaluate the fitness of the first order (2), modified Gompertz (3) and Chen and Hashimoto kinetic (4) models, the cumulative biogas data (sum of daily production) was plotted against time as presented in Figure 6. The results obtained from the models are presented respectively in Table 5, Table 6 and Table 7. All the models’ predictiveness were found to be significant with less than 5% deviation at 95% confidence level. Interestingly, all the kinetic models show that the addition of the Fe_3_O_4_ additives in bioreactor D increased the biogas production. This is because Fe_3_O_4_ addition reduced the detrimental effects of sulphides on methanogenesis by forming FeS precipitates. In addition, differences in lag phase times were observed for bioreactors A–E, while that of bioreactor D recorded the lowest at 15 days (Table 6) by the modified Gompertz model. This is explained by the fact that the microbial communities of bioreactor D were well adapted to their environment. Against the background of the AD process mechanism, the initial hydrolysed monomers and subsequent produced volatile fatty acids were rapidly consumed by the acidogenic and the methanogenic bacteria, respectively, with increased biogas production [50]. Among the applied kinetic models (Table 5, Table 6 and Table 7), the Gompertz model that predicted the cumulative biogas production with the smallest value (66–92 mL/day) had a perfect fit of regression coefficient (R^2^) within 0.9906–0.9939, affirming other reported studies [16,44].

## 4. Conclusions

The potential of three biomaterials (designated as CE, PB, and BI) as biostimulant additions for anaerobic digestion (AD) of municipal wastewater into biogas was investigated in this study in comparison to magnetite (Fe_3_O_4_). The additives demonstrated great potential for the abatement of high strength organic wastewater with more than 70% degree of reduction efficiency. Each additive had distinctive adsorptive pores as reflected by the SEM/EDX surface area obtained. This made the Fe_3_O_4_ with the highest surface area of 6 mm more advantageous for creating a broad pore size distribution than the rest (Fe_3_O_4_ > BI > CE > PB). In addition, the outcome of assessing the impact of the biosorbent on biogas production and AD process enhancement, conducted at 40 ℃ and HRT of 30 days was successful. Above all, the addition of 1.5 g of Fe_3_O_4_ nanoparticles assigned to bioreactor D maximised the average daily biogas (40 mL/day), as compared to CE (34 mL/day) > PB (3381 mL/day) > BI (32 mL/day) > control (25 mL/day). In addition, the modified Gompertz model was found to be appropriate for the prediction of biogas production as compared to the first order and Chen and Hashimoto kinetic models. The prospect of reinforcing this finding is to be encouraged, with optimised lab-scale and pilot scale plants on specified wastewater settings.

## Figures and Tables

**Table 1:** Biomaterials and magnetite used for wastewater remediation and energy production as compared to current study.

Biosorbent	Waste or Raw Material Used	Treatment Process	Results	Reference
Calcined banana Peels	Synthetic water prepared by diluting concentrated Mn(VII) and Fe(II) with deionised water	Adsorption	The biochar from banana peels was treated with pristine and phosphoric acid; the phosphoric acid pre-treatment had a better absorption efficiency than the pristine pre-treatment.	[40]
Raw Banana Peels	Automotive industrial wastewater Dirty water (river and rainwater)	Primary water treatment Water purification	The process had the highest removal of copper (93.52%) and lead (87.44%). The physical test met the quality conditions except for temperatures that exceeded the quality conditions of the maximum standard value.- Bacteriologically there were a lot of total coliforms exceeding the maximum standard conditions.	[24]
Egg shell	Real electroplating wastewaters containing Cr, Pb and Cd and synthetic wastewater containing heavy metals (Cr, Pb and Cd)	Jar-test coagulation process	The reuse of waste eggshell in the removal of toxic heavy metals, i.e., Cd and Cr in synthetic wastewater was much enhanced when calcined eggshell was added; however, removal of Pb was rather favourable with natural eggshell.	[23]
Fe_3_O_4_	Anaerobic sludge acquired from an Anaerobic-Anoxic-Oxic (AAO) reactor	Batch anaerobic digestion process	There was a 28% increase in biogas yield and COD removal of 14,760 mg/L in the reactor with Fe_3_O_4_	[41]
* Calcined banana Peels	Domestic and municipal wastewater	Biochemical methane potential (BMP) test	32.258 mL/day biogas yield, 73.53%, 71.05% and 88.93% COD, color and turbidity removal, respectively.	This study
* Raw Banana Peels	Domestic and municipal wastewater	BMP	33.226 mL/day biogas yield, 72.69%, 70.35% and 94.13% COD, color and turbidity removal, respectively	This study
* Egg shell	Domestic and municipal wastewater	BMP	32.581 mL/day biogas yield, 73.11%, 69.65% and 94.26% COD, color and turbidity removal, respectively.	This study
* Fe_3_O_4_	Domestic and municipal wastewater	BMP	37.807 mL/day biogas yield, 92.59%, 74.86% and 94.13% COD, color and turbidity removal, respectively.	This study

**Table 2:** Characteristics of wastewater and sludge samples.

Parameters	Results
Chemical oxygen demand (COD) (mg/L)	2380 ± 57.6
Color (Pt.Co)	57 ± 12.5
Turbidity (NTU)	17.32 ± 2.2
Total solids (TS) (mg TS/L)	204.5 ± 24.6
Volatile solids (VS) (mg VS/L)	106 ± 32.6
pH	6.59 ± 1.3

**Table 3:** The biosorbent loading for the BMP test.

Set-Up	Biosorbent Type	Biosorbent Loading (g)	Wastewater (mL)	Inoculum (mL)
A	Calcined banana peels (BI)	1.5	500	300
B	Crushed eggshell (CE)	1.5	500	300
C	Banana peels (PB)	1.5	500	300
D	Magnetite (Fe_3_O_4_)	1.5	500	300
E	Control (no loading)	n/a	500	300

**Table 4:** Average and cumulative biogas yield for HRT 30 days.

Bioreactor	Biosorbent Added (g)	Average Biogas Yield (mL/day)	Cumulative Biogas Yield (mL/day)
A	1.5	32	1000
B	1.5	34	1030
C	1.5	33	1010
D	1.5	40	1117
E	No additives	25	775

**Table 5:** Summary of the First order kinetic models for bioreactors A–E.

Set-Up	A	B	C	D	E
Yt (mL/g COD)	1 × 10^3^	1.03 × 10^3^	1.01 × 10^3^	1.17 × 10^3^	775
Ym (mL/g COD)	3.68 × 10^5^	2.06 × 10^5^	3.71 × 10^5^	2.96 × 10^5^	3.08 × 10^5^
k (1/day)	8 × 10^−4^	1.4 × 10^−4^	8 × 10^−4^	1.3 × 10^−4^	7 × 10^−4^
SSR	5.92 × 10^5^	7.44 × 10^5^	7.64 × 10^5^	4.57 × 10^5^	4.19 × 10^5^
R^2^	0.927	0.919	0.919	0.968	0.923
Predicted value (mL/g COD)	874	878	864	1.20 × 10^3^	655
Difference between measured (Yt) and predicted values	126	152	146	31	120

**Table 6:** Summary of the modified Gompertz kinetic models for bioreactors A–E.

Set-Up	A	B	C	D	E
Y(t) (mL/g COD)	1 × 10^3^	1.03 × 10^3^	1.01 × 10^3^	1.17 × 10^3^	775
Ym (mL/g COD)	1.89 × 10^3^	1.75 × 10^3^	1.57 × 10^3^	1.46 × 10^3^	1.40 × 10^3^
ʎ (days)	23.58	22.49	21.45	15.79	23.33
k (1/day)	0.081	0.094	0.106	0.123	0.884
SSR	3.79 × 10^5^	3.03 × 10^5^	2.71 × 10^5^	4.16 × 10^5^	1.64 × 10^5^
R^2^	0.991	0.993	0.994	0.993	0.993
Predicted value (mL/g COD)	1.09 × 10^3^	1.12 × 10^3^	1.09 × 10^3^	1.25 × 10^3^	841
Difference between measured (Yt) and predicted values (mL)	92	86	81	78	66

**Table 7:** Summary of the Chen and Hashimoto kinetic model for bioreactors A–E.

Set-Up	A	B	C	D	E
Yt (mL)	1 × 10^3^	1.03 × 10^3^	1.01 × 10^3^	1.17 × 10^3^	7.75 × 10^2^
Ym (mL)	5.33 × 10^5^	2.08 × 10^5^	4.12 × 10^5^	1.81 × 10^5^	1.82 × 10^5^
Rmax (mL/day)	3.6 × 10^−5^	2.2 × 10^−5^	1.9 × 10^−5^	3.7 × 10^−5^	0.16
K_CH_ (1/day)	6.73	1.59	0.28	1.71	2 × 10^−5^
SSR	5.92 × 10^5^	7.42 × 10^5^	7.65 × 10^5^	4.56 × 10^5^	4.21 × 10^5^
R^2^	9.28 × 10^−1^	9.19 × 10^−1^	9.2 × 10^−1^	9.69 × 10^−1^	9.24 × 10^−1^
Predicted value (mL/g COD)	876	880	864	1.20 × 10^3^	654
Difference between measured (Yt) and predicted values (mL)	124	150	164	31	121

## Data Availability

Not applicable.

## References

[1] Abdelwahab T.A.M., Mohanty M.K., Sahoo P.K., Behera D. (2020). Application of nanoparticles for biogas production: Current status and perspectives. Energy Sources Part A Recover. Util. Environ. Eff..

[2] Zaidi S.A.A., RuiZhe F., Shi Y., Khan S.Z., Mushtaq K. (2018). Nanoparticles augmentation on biogas yield from microalgal biomass anaerobic digestion. Int. J. Hydrogen Energy.

[3] Msibi S.S., Kornelius G. (2017). Potential for domestic biogas as household energy supply in South Africa. J. Energy S. Afr..

[4] Omara A.E.-D., Elsakhawy T., Alshaal T., El-Ramady H., Kovács Z., Fári M. (2019). Nanoparticles: A Novel Approach for Sustainable Agro-productivity. Environ. Biodivers. Soil Secur..

[5] Chollom M., Rathilal S., Swalaha F., Bakare B., Tetteh E. (2020). Removal of Antibiotics During the Anaerobic Digestion of Slaughterhouse Wastewater. Int. J. Sustain. Dev. Plan..

[6] Li X., Wang C., Zhang J., Liu J., Liu B., Chen G. (2020). Preparation and application of magnetic biochar in water treatment: A critical review. Sci. Total. Environ..

[7] Fermoso F.G., van Hullebusch E., Collins G., Roussel J., Mucha A.P., Esposito G. (2019). Trace Elements in Anaerobic Biotechnologies.

[8] Tetteh E.K., Rathilal S. (2020). Kinetics and Nanoparticle Catalytic Enhancement of Biogas Production from Wastewater Using a Magnetized Biochemical Methane Potential (MBMP) System. Catalysts.

[9] Ángeles R., Vega-Quiel M.J., Batista A., Fernández-Ramos O., Lebrero R., Muñoz R. (2021). Influence of biogas supply regime on photosynthetic biogas upgrading performance in an enclosed algal-bacterial photobioreactor. Algal Res..

[10] Nethengwe N.S., Uhunamure S.E., Tinarwo D. (2018). Potentials of biogas as a source of renewable energy: A case study of South Africa. Int. J. Renew. Energy Res..

[11] Budzianowski W.M., Postawa K. (2017). Renewable energy from biogas with reduced carbon dioxide footprint: Implications of applying different plant configurations and operating pressures. Renew. Sustain. Energy Rev..

[12] Michailos S., Walker M., Moody A., Poggio D., Pourkashanian M. (2020). Biomethane production using an integrated anaerobic digestion, gasification and CO_2_ biomethanation process in a real waste water treatment plant: A techno-economic assessment. Energy Convers. Manag..

[13] Hülsemann B., Zhou L., Merkle W., Hassa J., Müller J., Oechsner H. (2020). Biomethane Potential Test: Influence of Inoculum and the Digestion System. Appl. Sci..

[14] Lohani S.P., Havukainen J. (2018). Anaerobic Digestion: Factors Affecting Anaerobic Digestion Process.

[15] Rehman M.L.U., Iqbal A., Chang C., Li W., Ju M. (2019). Anaerobic digestion. Water Environ. Res..

[16] Sarto S., Hildayati R., Syaichurrozi I. (2019). Effect of chemical pretreatment using sulfuric acid on biogas production from water hyacinth and kinetics. Renew. Energy.

[17] Chen C., Guo W., Ngo H.H., Lee D.-J., Tung K.-L., Jin P., Wang J., Wu Y. (2016). Challenges in biogas production from anaerobic membrane bioreactors. Renew. Energy.

[18] Hagos K., Zong J., Li D., Liu C., Lu X. (2017). Anaerobic co-digestion process for biogas production: Progress, challenges and perspectives. Renew. Sustain. Energy Rev..

[19] Yoshida K., Shimizu N. (2020). Biogas production management systems with model predictive control of anaerobic digestion processes. Bioprocess Biosyst. Eng..

[20] Xu D., Ji H., Ren H., Geng J., Li K., Xu K. (2020). Inhibition effect of magnetic field on nitrous oxide emission from sequencing batch reactor treating domestic wastewater at low temperature. J. Environ. Sci..

[21] Thanh P.M., Ketheesan B., Yan Z., Stuckey D. (2016). Trace metal speciation and bioavailability in anaerobic digestion: A review. Biotechnol. Adv..

[22] Luna-Delrisco M., Orupõld K., Dubourguier H.-C. (2011). Particle-size effect of CuO and ZnO on biogas and methane production during anaerobic digestion. J. Hazard. Mater..

[23] Goli J., Sahu O. (2018). Development of heterogeneous alkali catalyst from waste chicken eggshell for biodiesel production. Renew. Energy.

[24] Sridhar N., Senthilkumar J.S., Subburayan M.R. (2014). Removal of Toxic Metals (Lead & Copper) From Automotive Industry Waste Water By Using Fruit Peels. Int. J. Adv. Inf. Commun. Technol..

[25] Amo-Duodu G., Rathilal S., Chollom M.N., Tetteh E.K. (2021). Application of metallic nanoparticles for biogas enhancement using the biomethane potential test. Sci. Afr..

[26] Hassanein A., Keller E., Lansing S. (2021). Effect of metal nanoparticles in anaerobic digestion production and plant uptake from effluent fertilizer. Bioresour. Technol..

[27] Chen L., Feng W., Fan J., Zhang K., Gu Z. (2020). Removal of silver nanoparticles in aqueous solution by activated sludge: Mechanism and characteristics. Sci. Total Environ..

[28] Toor R., Mohseni M. (2007). UV-H2O2 based AOP and its integration with biological activated carbon treatment for DBP reduction in drinking water. Chemosphere.

[29] Pirilä M. (2015). Adsorption and Photocatalysis in Water Treatment: Active, Abundant and Inexpensive Materials and Methods.

[30] Lakshmanan R. (2013). Application of Magnetic Nanoparticles and Reactive Filter Materials for Wastewater Treatment Ramnath Lakshmanan. Ph.D. Thesis.

[31] Zhang Y., Wu B., Xu H., Liu H., Wang M., He Y., Pan B. (2016). Nanomaterials-enabled water and wastewater treatment. NanoImpact.

[32] Qiu X., Zhang Y., Zhu Y., Long C., Su L., Liu S., Tang Z. (2021). Applications of Nanomaterials in Asymmetric Photocatalysis: Recent Progress, Challenges, and Opportunities. Adv. Mater..

[33] Maksoud M.I.A.A., Elgarahy A., Farrell C., Al-Muhtaseb A.H., Rooney D.W., Osman A. (2020). Insight on water remediation application using magnetic nanomaterials and biosorbents. Coord. Chem. Rev..

[34] Peeters K., Lespes G., Zuliani T., Ščančar J., Milačič R. (2016). The fate of iron nanoparticles in environmental waters treated with nanoscale zero-valent iron, FeONPs and Fe_3_O_4_NPs. Water Res..

[35] Tetteh E.K., Amankwa M.O., Armah E.K., Rathilal S. (2020). Fate of COVID-19 Occurrences in Wastewater Systems: Emerging Detection and Treatment Technologies—A Review. Water.

[36] Lombi E., Donner E., Tavakkoli E., Turney T., Naidu R., Miller B.W., Scheckel K. (2012). Fate of Zinc Oxide Nanoparticles during Anaerobic Digestion of Wastewater and Post-Treatment Processing of Sewage Sludge. Environ. Sci. Technol..

[37] Adetunji A.I., Olaniran A.O. (2021). Treatment of industrial oily wastewater by advanced technologies: A review. Appl. Water Sci..

[38] Tetteh E.K., Rathilal S., Chetty M., Armah E.K., Asante-Sackey D. (2019). Treatment of Water and Wastewater for Reuse and Energy Generation-Emerging Technologies.

[39] Gunnerson C., Stuckey D. (1986). Integrated Resource Recovery-Anaerobic Digestion.

[40] Hossain M.A. (2012). Removal of Copper from Water by Adsorption onto Banana Peel as Bioadsorbent. Int. J. Geomate.

[41] Xiang Y., Yang Z., Zhang Y., Xu R., Zheng Y., Hu J., Li X., Jia M., Xiong W., Cao J. (2019). Influence of nanoscale zero-valent iron and magnetite nanoparticles on anaerobic digestion performance and macrolide, aminoglycoside, β-lactam resistance genes reduction. Bioresour. Technol..

[42] Adeleke O., Akinlabi S., Jen T.-C., Dunmade I. (2021). Towards sustainability in municipal solid waste management in South Africa: A survey of challenges and prospects. Trans. R. Soc. S. Afr..

[43] APHA (2012). Standard Methods for the Examination of Water and Wastewater.

[44] Syaichurrozi B.I., Sumardiono S. (2014). Kinetic Model of Biogas Yield Production from Vinasse at Various Initial pH: Comparison between Modified Gompertz Model and First Order Kinetic Model. Res. J. Appl. Sci. Eng. Technol..

[45] Mu Y., Wang G., Yu H.-Q. (2006). Kinetic modeling of batch hydrogen production process by mixed anaerobic cultures. Bioresour. Technol..

[46] Sibiya N.P., Rathilal S., Tetteh E.K. (2021). Coagulation Treatment of Wastewater: Kinetics and Natural Coagulant Evaluation. Molecules.

[47] Goswami R.K., Mehariya S., Karthikeyan O.P., Verma P. (2020). Advanced microalgae-based renewable biohydrogen production systems: A review. Bioresour. Technol..

[48] Pessoa M., Sobrinho M.M., Kraume M. (2020). The use of biomagnetism for biogas production from sugar beet pulp. Biochem. Eng. J..

[49] Abdelradi F. (2018). Food waste behaviour at the household level: A conceptual framework. Waste Manag..

[50] Baek G., Kim J., Cho K., Bae H., Lee C. (2015). The biostimulation of anaerobic digestion with (semi)conductive ferric oxides: Their potential for enhanced biomethanation. Appl. Microbiol. Biotechnol..

